# Skin Photodamage Lesions in a Bilateral Feline Auricular Primary Fibrosarcoma

**DOI:** 10.3390/vetsci9100548

**Published:** 2022-10-05

**Authors:** Francesca Parisi, Francesca Abramo, Marco Maimone, Alessandro Poli, Francesca Millanta

**Affiliations:** 1 Department of Veterinary Sciences, University of Pisa, Viale delle Piagge n. 2, 56124 Pisa, Italy; 2 Clinica Veterinaria Foce, via Eugenio Baroni, 26R, 16129 Genova, Italy

**Keywords:** auricular, cats, elastosis, fibrosarcoma, solar, UV light

## Abstract

**Simple Summary:**

Recent studies suggest a photoinduced etiopathology not only for actinic keratosis and squamous cell carcinomas, but also for non-epithelial cutaneous tumors in feline species. We report a recent case of a ten-year-old male cat with a white-hair coat and mesenchymal neoplasms of both auricles. Cytology, complete blood count (CBC), serum biochemistry and imaging examinations were performed. After surgery, the samples underwent routinary histopathology and special staining with orcein. A routine analysis yielded values within a normal range and X-rays and ultrasonography showed no abnormalities. The cytology was inconclusive, but, through histopathology, two well-differentiated fibrosarcomas were diagnosed and histopathological changes related to chronic UV exposure were documented in the skin close to the lesions. An orcein stain highlighted elastosis, the hallmark of photodamage; a morphometric analysis showed that the elastotic material was more abundant in the dermis close to the tumors. Therefore, an involvement of UV rays in the carcinogenic process of these tumors may be suspected.

**Abstract:**

As with human species, recent studies also suggest a photoinduced etiopathology for non-epithelial cutaneous tumors in feline species. We report a recent case of a ten-year-old male cat with a white-hair coat and mesenchymal neoplasms of both auricles. Cytology, complete blood count (CBC), serum biochemistry and imaging examinations were performed. After surgery, the samples underwent routinary histopathology and were additionally stained with orcein. A routine analysis yielded values within a normal range and the imaging examination showed no abnormalities, suggesting that the bilateral presentation of neoplasms was primary rather than metastatic. The cytology was inconclusive, but, through histopathology, two well-differentiated fibrosarcomas were diagnosed and histopathological changes related to chronic UV exposure (such as epidermal hyperplasia, stratification disorders, keratinocyte dysplasia and an accumulation of elastotic material) were documented in the skin adjacent to the lesions. An orcein stain succeeded in highlighting elastosis. The elastic fibers lost their regular structure and orientation and appeared to be fragmented, wavy to branched and knotted. A morphometric analysis showed that the amount of elastotic material in the dermis close to the tumors was more than double compared with the more distant areas. Elastosis is considered to be a hallmark of photodamage; thus, an involvement of UV rays in the carcinogenic process of the tumors may be suspected.

## 1. Introduction

A causal role of UV radiation in the carcinogenesis of skin cancers has been confirmed at least from 1992, when the International Agency for Research on Cancer stated that “There is sufficient evidence in humans for the carcinogenicity of solar radiation. Solar radiation causes cutaneous malignant melanoma and non- melanocytic skin cancer” [1]. All the evidence for the carcinogenicity of solar and ultraviolet radiation that has accumulated since 1992 has strengthened this conclusion. Nowadays, in human medicine, there are sufficient data that at least cutaneous malignant melanomas and epidermal tumors such as basal cell carcinomas (BCC) and squamous cell carcinomas (SCC) of the skin (also known, collectively, as non-melanocytic skin cancer) are caused by exposure to solar radiation [2]. However, there are more and more case reports about a probable involvement of UV rays also in the carcinogenic process of other types of cutaneous tumors. Particularly, a contribution of UV light was suggested in the development of human melanomas [3,4], cutaneous dermal sarcomas [5,6,7,8] and vascular tumors [9].

In the veterinary literature, as with humans, several cases of canine cutaneous hemangiomas [10,11,12], canine conjunctival hemangiomas and hemangiosarcomas [13] as well as equine ocular, periocular and vulvar hemangiosarcomas [14,15] were suspected to be linked in some way to UV radiation exposure. The case report described here has already been involved in a recent study of Millanta et al., who dealt with five cases of feline non-epithelial cutaneous tumors with histopathological changes characteristic of chronic solar irradiation and, particularly, with elastosis [16], an important and recognized sign of photodamage [14,17,18,19,20]. In this paper, we aim to describe in more detail the histopathological features indicative of skin photodamage in the skin adjacent to a bilateral auricular primary fibrosarcoma in a domestic short-hair cat, together with the clinical history and laboratory and imaging diagnostic findings. Given the few studies about cutaneous non-epithelial tumors, the correlation between UV exposure and the occurrence of these tumors has not yet been proven; further studies are needed to clarify this aspect.

## 2. Case Description

**Figure 1:**
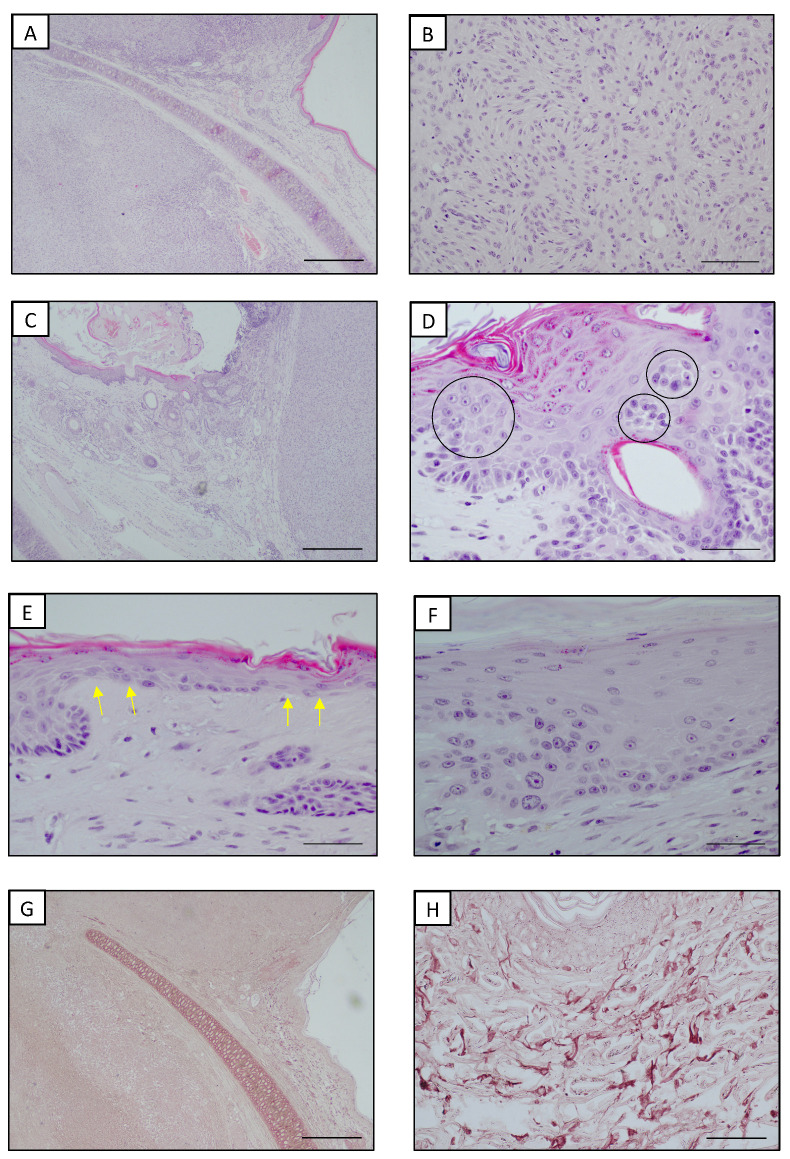
Auricular pinna, cat. (**A**) Presence of well-circumscribed neoplasms deep in the dermis. Hyperplastic epidermis was present in tissues adjacent to the neoplasm. HE. Bar = 500 μm. (**B**) Fibrosarcoma. Intermediate-sized spindle cells arranged into storiform and vortex architectures. HE. Bar = 100 μm (**C**) Hyperplastic epidermis with stratification disorders at the periphery of a well-circumscribed fibrosarcoma. HE. Bar = 500 μm. (**D**) Higher magnification of hyperplastic epidermis with stratification disorders: proliferation nests (black circles). HE. Bar = 50 μm. (**E**) Higher magnification of hyperplastic epidermis with stratification disorders: squamatization of the basal layer (yellow arrows). HE. Bar = 50 μm. (**F**) Higher magnification of hyperplastic epidermis with stratification disorders: dysplastic keratinocytes in the basal layer. HE. Bar = 50 μm. (**G**) Superficial dermis adjacent to the neoplasm showed elastosis. Orcein staining. Bar = 500 μm. (**H**) Higher magnification of thickened, hypertrophic, fragmented and branched elastic fibers. Orcein staining. Bar = 100 μm.

A 10-year-old castrated male domestic short-hair cat with a white coat was presented with two lesions on both convex surfaces of the auricular pinnae. According to the owners, the cat lived both indoors and outdoors, spending a considerable amount of time in the garden. Upon a physical examination, the cat was alert, showed a good body condition (a body condition score of 5 on a scale from 1 to 9) and the vital signs were normal. Notably, the regional lymph nodes were normal as well. A dermatological examination showed that these neoplasms were two well-circumscribed and firm nodules, approximately 1.5 × 1.0 cm and 0.8 × 0.5 cm; both were alopecic with an overlaying crust. The clinical differential diagnoses included infective lesions and primary or metastatic neoplastic disease. A cytological specimen was obtained by an impression smear technique after lightly scraping the crusts. Slides stained with Diff Quick (Dif Stain Kit **^®^** Titolchimica, Rovigo, Italy) showed a predominance of clusters of medium-sized epithelial cells with round to oval nuclei and a faded cytoplasm, together with very few mesenchymal cells with oval nuclei and basophilic cytoplasm, several of them with caudated processes. No inflammatory cells or etiological agents were noticed. Overall, the cells did not show severe cytologic atypia or other cytologic features suggestive of a malignancy. Through the cytological examination, an infectious etiopathogenesis was excluded but it was not possible to obtain a certain diagnosis. Routine laboratory testing revealed complete blood count (CBC) and serum biochemistry within normal reference intervals, aside from mild hyperglycemia (129.0 mg/dL; reference range 58.0–120.0 mg/dL), probably due to the clinical manipulations, and a small increase in GGT (11.0 µL/L; reference range 0.0–2.0 µL/L). The cat tested negative for feline immunodeficiency virus (FIV) and feline leukemia virus (FeLV). Before surgery, no abnormalities were found on a lateral radiograph of the thorax and abdomen or abdominal ultrasonography. Based on these findings, a primary bilateral auricular neoplasm was suspected. The surgical removal of the lesion was thus proposed by a total conchectomy for safety margins and a subsequent histopathological examination. The cat was premedicated with medetomidine (Sedastart**^®^** Esteve 20 µg/kg) and methadone (Semfortan**^®^** Dechra 0.3 mg/kg) and induced with propofol (Proposure**^®^** Merial 1 mg/kg). The anesthesia was maintained with isoflurane (Vetflurane; Virbac) in oxygen. Both surgically removed nodular masses were fixed in 10% neutral buffered formalin and routinely processed. The histopathological examination of the nodules (hematoxylin and eosin stain (HE)) revealed the presence of two not well-demarcated and not capsulated nodular masses in the dermis, covered by focally ulcerated and crusted skin (Figure 1A). The two neoplastic masses were a nodular proliferation of fibroblast-like cells with elongated, oval or spindle-shaped nuclei, a scant cytoplasm and one or more prominent nucleoli. The neoplastic cells were organized in interlaced bundles interspersed by collagen fibers (Figure 1B). According to the grading system for cutaneous and subcutaneous soft tissue sarcomas in cats proposed by Dobromylskyi et al. [21], the two fibrosarcomas were classified as two intermediate grade fibrosarcomas. One fibrosarcoma particularly showed a very mild necrosis located under the ulcerated epithelial surface, mild to moderate inflammation and 17 mitoses per 10 high power fields (2.37 mm^2^). On the contrary, necrosis and inflammation were absent in the contralateral fibrosarcoma, which was characterized by 24 mitoses within the defined area of 2.37 mm^2^. Moderate UV-induced alterations such as epidermal hyperplasia and stratification disorders (e.g., the presence of proliferation nests, the squamatization of the basal layer and keratinocyte dysplasia) were observed in the epidermis above and around the masses; moreover, abundant elastotic material was accumulated in the dermis close to the tumors (Figure 1C–E). To confirm and better detail elastosis, orcein staining was performed to highlight solar elastosis following the manufacturer’s instructions (Orcein Kit**^®^**, Bio-Optica, Milano, Italy). The qualitative analysis of the slides stained with orcein showed abnormal elastic fibers that were losing their regular structure and orientation. The elastic fibers in the superficial dermis appeared thickened and fragmented, with a wavy to branched jagged pattern that was sometimes knotted (Figure 1F,G).

**Figure 2:**
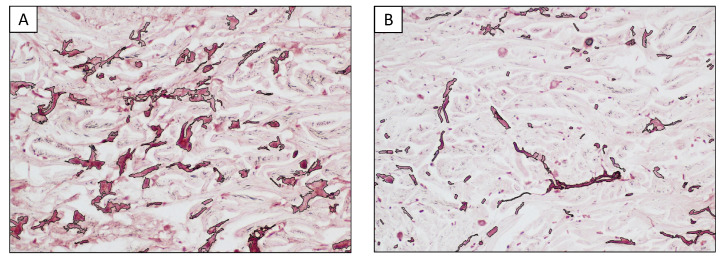
Representative example of morphometric analysis. The area occupied by elastotic material was assessed for each field over a total area of 0.27 mm^2^. (**A**) Dermis adjacent to the lesions. (**B**) Dermis far from the lesions in a less UV-exposed area.

A morphometric comparative analysis of the elastotic material was carried out to compare the areas adjacent to the lesions as well as in the convex surface of the pinna and areas far from the tumors in the less UV-exposed concave surface (Charoenchon et al., 2018, modified [22]). In detail, five areas in the uppermost layer of the epidermis adjacent to the neoplasm and five distant from the lesion were captured, extending to a depth of 200 µm from the dermal epidermal junction. The area occupied by the orcein-positive elastotic material was assessed using a 40 × objective in a total area of 0.27 mm^2^ for each of the ten regions of interest, using dedicated software for computer-assisted image analyses (NIS-Elements Br Microscope Imaging Software, Nikon Instruments; Calenzano, Italy). A morphometric comparative analysis showed that the amount of orcein-positive elastotic material in the dermis close to the tumors was more than double compared with the distant UV-exposed areas (7.4% vs. 3.1%; Figure 2). This result suggested that not only was the dermis adjacent to the fibrosarcomas characterized by abnormal elastic material, but also that this material was more abundant in these districts when compared with those far from the neoplastic lesions.

One and six months after surgery, the clinical condition of the cat was unremarkable at the follow-up examinations. The hematological and biochemical values were within normal ranges. No signs of local recurrences were observed.

## 3. Discussion

To our knowledge, this is the first case report of a bilateral feline auricular primary fibrosarcoma associated with photodamage lesions. The role of UV radiation in the development of feline squamous cell carcinomas and their preneoplastic precursor lesions, actinic keratosis, has already been clearly proven [23,24]. Only recently, an analogous etiopathogenesis was also suggested for feline cutaneous non-epithelial tumors, particularly those arising in areas of the body directly exposed to UV rays such as, as in this case, the auricular pinnae. Unlike dogs, cats rarely lie in dorsal recumbence and their ventral hair is thicker, so UV-induced lesions occur almost exclusively on the head [17].

Important findings suggesting the hypothesis of an UV-ray involvement in certain types of tumors are the association between lesions and concomitant histopathological changes that universally correlate with photoaging in humans [25,26,27] and in veterinary medicine [28,29,30]. In this case report, the areas adjacent to the two tumors showed photoinduced changes such as epidermal hyperplasia, epithelial stratification disorders, the squamatization of the basal layer and the presence of proliferation nests of keratinocytes. The most important finding was the presence of elastosis, highlighted through orcein staining, as an accumulation of abnormal, fragmented and knotted basophilic elastic fibers in the dermis that were losing the thin linear pattern typical of normal elastic fibers found in healthy skin. In mice, the hyperplasia of elastic fibers is one of the earliest signs of photoaging, followed by the degeneration of the elastin matrix and massive quantities of thickened, degraded fibers that accumulate when an ultraviolet (UV) assault persists, eventuating in elastosis [20]. In humans, solar elastosis is commonly associated with photoaging because elastic fibers become more abundant and fragmented after excessive exposure to UV radiation [31,32,33]. Furthermore, the degree of elastosis in humans correlates with the amount of UV rays to which the skin is exposed [34]. Even if a few authors have suggested that solar elastosis is a rare manifestation of solar-induced lesions in cats due to the small number of elastic fibers [17], the more recent study of Millanta et al., which also included the case described herein, highlighted the presence of elastosis in the dermis of cats with non-epithelial cutaneous tumors arising in the auricular pinnae, suggesting an involvement of UV radiation in the development of those neoplasms [16]. An orcein stain was useful in those cases to demonstrate the elastic fiber changes. The validation of solar elastosis grading is of great relevance to both human and animal dermatology; in human medicine, many papers have attempted this difficult aim by suggesting different grading schemes [35,36,37]. To date, there are no similar grading systems proposed in the veterinary literature and, due to the species differences, the human grading may not be suitable for animals. Thus, we used morphometry to quantify the coverage of the orcein-stained elastotic material in an area corresponding with the human papillary dermis, which we considered to be more useful than providing a subjective estimation of the degree of damage. Moreover, the data obtained per mm^2^ of tissue were expressed as a percentage; this will allow comparisons with further new studies, avoiding possible misleading due to species differences.

The auricular pinnae have already been reported as a preferential site for dermal fibrosarcomas in older cats [30], but the presence of multiple lesions arising in a background of histopathological changes commonly associated with photodamage and, above all, of elastosis (commonly considered a hallmark of photodamage) may suggest a photoinduced pathogenesis in this case. Moreover, the clinical case herein supported a primary bilateral involvement of the neoplasm in the auricular pinnae, testified by unremarkable physical, X-ray and abdominal ultrasound examinations. The CBC and serum biochemistry showed only mild alterations of glycemia, probably due to the stress of the manipulations, and a mild increase in GGT, which was considered to be irrelevant because it was not associated with further signs of hepatic or systemic involvements. Moreover, during the one-month-later control, the CBC and serum biochemistry showed all values within a normal range. 

Cytology was useful to eliminate a suspicion of an infectious etiopathogenesis; it was considered to be inconclusive for a clinical suspicion of neoplasia because few cells with little signs of malignancy were observed and most of them were representative of the superficial layers. It is generally known that cytology is a less reliable diagnostic than histology; moreover, the sampling further affects the reliability of the diagnosis. In this case, the sampling was carried out through apposition after a gentle scraping due to the pain of the patient and to reduce the manipulation stress. A fine-needle aspiration of the mass would have been the method of choice to obtain a more reliable result; thus, it was not surprising that the cytology was not diagnostic. It is known that the fine-needle aspiration of mesenchymal tumors succeeds in sampling very few cells, so it was conceivable that in any case the cytology would not have been so useful in achieving a diagnosis. 

Beyond the pathological findings, the hypothesis of UV-ray involvement could also be plausible in this case because the patient had access to a garden, had a white coat and the owner reported that he liked sun baths. In humans, it has also been suggested that the risk increases with the accumulation of hours of sunlight from year to year [2]; feline lesions related to UV rays were reported to worsen with each passing summer [17]. The adult age of the cat may argue in favor of this hypothesis, and it was in line with the already existent literature on UV-induced lesions in humans and veterinary medicine [10,13,14,23,24], even if it has also been reported that younger cats could be affected due to more intense exposure to sun [17]. Specifically, the median age of cats reported with non-epithelial cutaneous tumors from the previous study of Millanta et al. was 8.4 years old [16]. Moreover, chronic solar-induced lesions occur most commonly in white-faced, blue-eyed cats [17] without a breed predilection [38]. UV-induced lesions are reported to be more common in sparsely pigmented subjects, probably due to the protective role of melanin in absorbing and scattering detrimental UV radiation [26].

The suspicion of a hypothetical involvement of UV radiation in the development of the bilateral fibrosarcoma was based on the presence of histopathological changes related to solar radiation, on the bilateral presentation of the lesion and on the fact that a white-haired cat is likely to become a victim, with the confirmation from the owners that the cat spent time outdoors sunbathing. Despite this, a conclusion about the relationship between UV harm and the development of these neoplasms could not be drawn based on the existence of these findings alone because the onset timing of the UV-related histopathological changes could not be determined. The only way to confirm a causal relationship would have been to identify a genomic fingerprint that could prove it, as with human studies. In the literature regarding humans, the genomic background of the most common skin tumors and their molecular mechanisms of photocarcinogenesis are well-known [39,40,41]. On the contrary, the veterinary literature is still lacking on this topic.

We consider this case interesting because studies on the possible role of UV light on non-epithelial cutaneous neoplasms and their possible prognostic significance could be relevant in veterinary as well as in comparative medicine because domestic animals, particularly cats, closely share the environment with their owners, acting as a sentinel for human environmental carcinogenic agents [17,42] and a model of human neoplasms [43].

Furthermore, skin neoplasms are often underestimated in common human ideology, as testified by a study from 2018 that reported a low level of concern of medical students regarding the perceived importance of skin cancer in comparison with other cancer types and rarely employed preventive measures [44]. If this is already true in human medicine, whose literature is full of studies on UV-induced neoplasms, it might be conceivable that there will be even less consciousness in veterinary medicine. Thus, the effort to research this field could lead to greater awareness, more focused education and more effective direct future intervention and prevention efforts, both in human and veterinary medicine.

## 4. Conclusions

This clinical case emphasizes the significance of considering a potential role of UV radiation in the etiopathogenesis of skin neoplasms. As a result, it is important to document the epidermal and dermal signs of solar-induced damage and to include specific staining that highlights elastin fibers such as the orcein staining used in this study or immunohistochemical staining to detect fibrillin-1, fibulin-2 and fibulin-5, as already conducted in the literature regarding humans [22], in order to highlight elastosis. In addition, collecting samples suitable for molecular studies is strongly recommended because only genomics can help to ascertain a UV-induced hypothesis. 

## Data Availability

Not applicable.
